# Acknowledgement to Reviewers of *Diagnostics* in 2013

**DOI:** 10.3390/diagnostics4010012

**Published:** 2014-02-28

**Authors:** Diagnostics Editorial Office

**Affiliations:** MDPI AG, Klybeckstrasse 64, CH-4057 Basel, Switzerland

The editors of *Diagnostics* would like to express their sincere gratitude to the following reviewers for assessing manuscripts in 2013:

Bolondi, Luigi
Boon, Elles M.j.
Breydo, Leonid
Caudle, Michael
Chang, Wei-chiao
Chitty, Lyn
Choolani, Mahesh
Comhaire, Frank
Cosgrove, David
Dacher, Jean-nicolas
Dahdah, Nagib
Daneshmand, Siamak
Eftratiou, Androulla
Egi, Moritoki
Fernanda, Falcini
Fox, Susan H.
Ghoshal, Kalpana
Gilbert, Peter B.
Giubellino, Alessio
Graves, Edward
Hafeez, Shaista
Hagen, Gaute
Hanson, Kim
Hidalgo-Grass, Carlos
Iqbal, Samir
Jackson, Dr. Graham S.
Kambadakone, Avinash
Keenan, D. Barry
Kim, Joon-ho
Kim, Min Jung
Kim, Young-kee
Kwee, Thomas C.
Lee, Junghwa
Leidner, Bertil
Li, Qiang
Lu, Yujie
Mercuri, Nicola Biagio
Meredith, Ruby F.
Modlin, Irvin
Moeller, Soeren
Moise, Kenneth J.
Natarajan, Sathish
Nuutila, Pirjo
Ohuchida, Kenoki
Ombelet, Willem
Pertl, Barbara
Petitjean, Caroline
Pollock, Nira
Satoh, Asami
Schäfer, Helmut
Schneider, Jurgen
Sendra, Francisco
Stojanovic, Milan N.
Themelis, George
Tsuji, Yoshihisa
Vazquezbenitez, Gabriela.x.
Wang, Jiahui
Welling, Mick
White, Bruce A.
Wong, Tak-sing
Wu, Jen-leih
Wu, Jian
Wu, Jie
Yang, Yongfeng
Zhou, Qian
Zlotta, Alexandre R.

